# Severe generalized edema in a premature neonate: A case report and literature review

**DOI:** 10.1002/ccr3.9341

**Published:** 2024-09-03

**Authors:** Haifeng Zong, Yingsui Huang, Ying Xiong, Wentao Gong, Bingchun Lin, Chuanzhong Yang

**Affiliations:** ^1^ Neonatal Intensive Care Unit Shenzhen Maternity and Child Healthcare Hospital Shenzhen China

**Keywords:** edema, hydrops, MAPK, newborn, Noonan syndrome, PTPN11

## Abstract

**Key Clinical Message:**

With no family history, and an atypical phenotype, the clinical diagnosing of Noonan syndrome (NS) can be very difficult. The present case emphasized that generalized edema in neonates may be the potential first symptom of NS.

**Abstract:**

Severe generalized edema is a rare pathological condition with high mortality in newborns, in particular the premature infants. It is characterized by the extensive subcutaneous tissue edema and the accumulation of fluid in neonatal body fluid compartments. The etiology and pathogenesis of hydrops in neonates are quite complex. Generally speaking, hydrops can be divided into immune hydrops and non‐immune hydrops according to the etiology. It is still challenging in treating severe neonatal edema. In this study, we presented a preterm newborn with severe generalized edema after birth, which was finally diagnosed with Noonan syndrome (NS). The infant clinically manifested as severe generalized edema alone, without the involvement of multiple organ malformation. Generalized edema in neonates was probably the first symptom of NS. Therefore, differential diagnosis of NS is necessary for infants developing generalized edema.

## 1 INTRODUCTION

Noonan syndrome (NS) accounts for a frequently seen, mainly autosomal‐dominant genetic disease, and its phenotype differs with regard to the severity, with the possible involvement of multi‐organ systems. Its incidence is estimated to be 1/1000–2500 live births.^1^ A de novo pathogenic variant can be detected in most infants, but 20%–40% of these patients are associated with their affected parents.^2^ NS mainly shows the features of dysmorphic facial characteristics, congenital heart diseases (usually pulmonary valve stenosis, atrial septal defects, and hypertrophic cardiomyopathy), proportionate post‐natal short stature, chest deformity, or neck abnormalities.^3^ The male‐to‐female ratio of NS is nearly 1:1.^3^ The present work reported a rare premature newborn with severe generalized edema being the first symptom.

## 2 CASE REPORT

### 2.1 Case history

**FIGURE 1:**
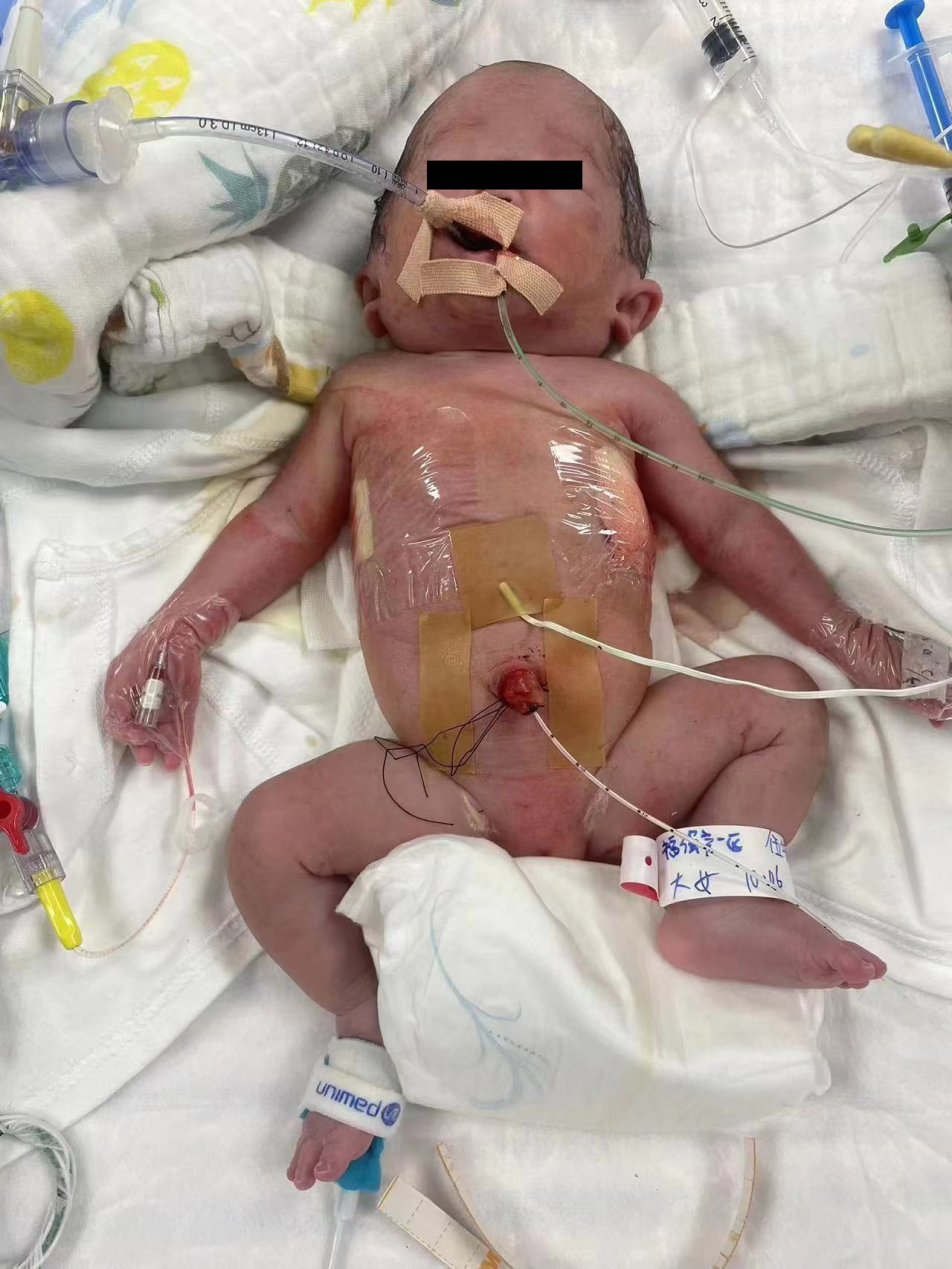
Photograph showing facial features of the neonate (low‐set ears, widely spaced eyes).

A female neonate (weight, 1510 g) was born at 30^+6^ weeks' gestation through cesarean section due to severe fetal distress. The Apgar scores at 1, 5, and 10 min were 3, 6, and 6 separately. This infant, in twin pregnancy, was born using in vitro fertilization (IVF). Deepest vertical pocket was 8.28 cm at 29^+6^ weeks of gestation. Extensive subcutaneous tissue edema, pleural effusion and seroperitoneum were diagnosed at 27 weeks of gestation. Invasive prenatal amniocentesis was performed to obtain fetal tissues for genetic analysis on 30^+4^ weeks' gestation. While the other fetus of the twin was diagnosed with intrauterine growth restriction. Upon physical examination (Figure 1), unique facial features, including low‐set ears and widely‐spaced eyes, were found. The infant also exhibited the respiratory distress symptoms. Auscultation both lungs revealed quiet breath sounds. The infant was intubated in the operating room due to neonatal asphyxia, and was transferred to the neonatal intensive care unit successfully after resuscitation.

### 2.2 Methods

**FIGURE 2:**
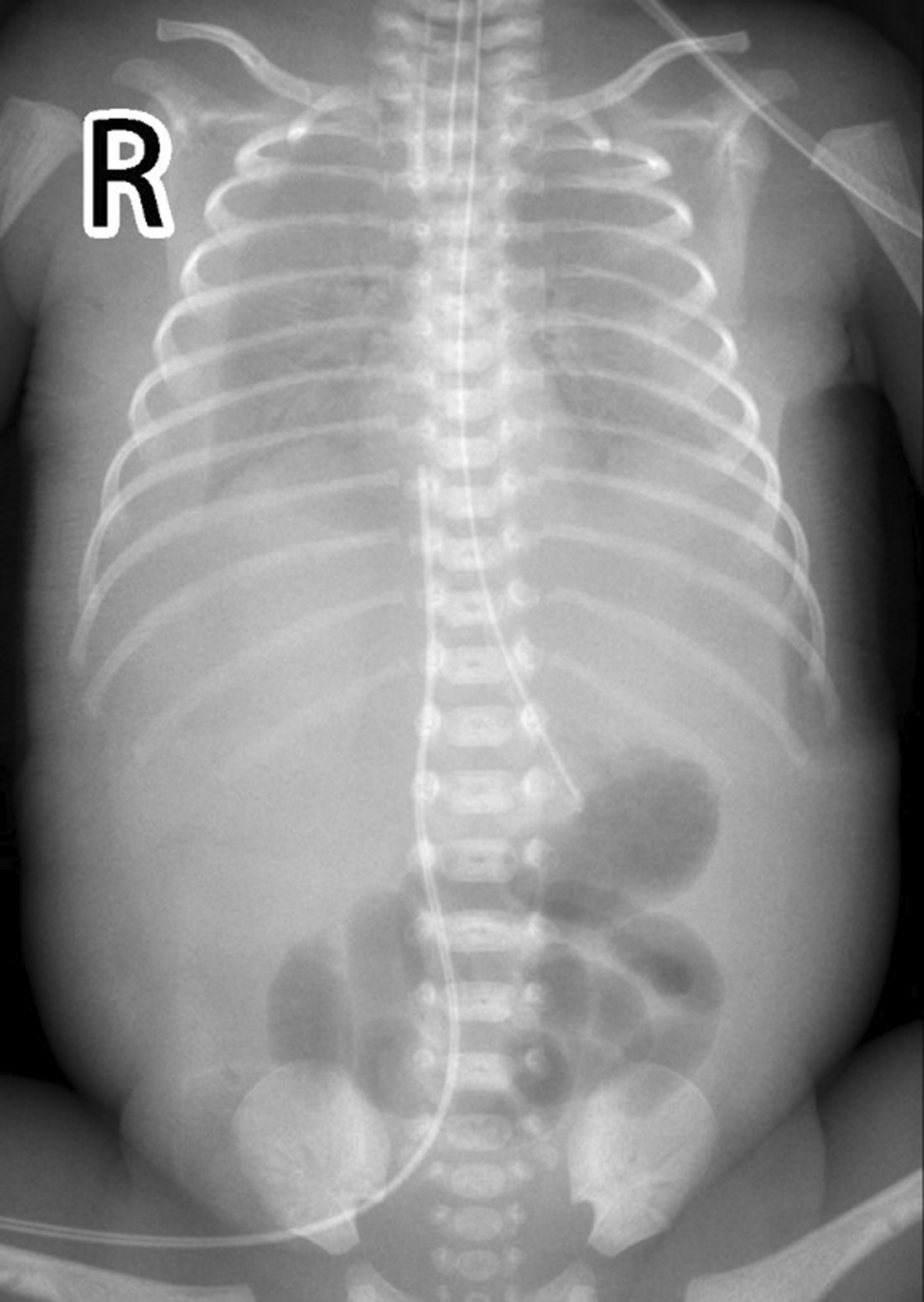
Chest x‐ray shows accumulation of pleural effusion with decreased lung volume.

**FIGURE 3:**
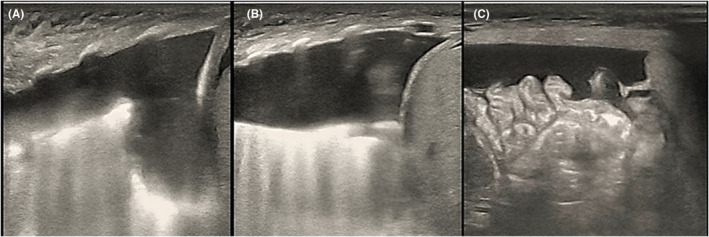
Ultrasound showed pleural effusion and seroperitoneum. (A) Left‐sided pleural effusion. (B) Right‐sided pleural effusion. (C) Seroperitoneum.

This newborn was given the diagnosis of neonatal respiratory distress syndrome (NRDS), so mechanical ventilation was administered immediately. Arterial blood gas analysis revealed PH 7.01, PaCO_2_ 89 mmHg, lactate 3.6 mmol/L, and HCO_3_ 22.5 mmol/L. Thoracentesis was performed with thoracic puncture and thoracic drainage, after which, the condition of this newborn became stable. Serum albumin level was 15 g/L, and infusion of human albumin was applied soon after birth. Serum alanine aminotransferase (ALT) level was 9 U/L, aspertate aminotransferase (AST) level was 67 U/L, and creatine kinase isoenzyme MB (CK‐MB) level was 2.4 ng/mL. The complete blood count showed that the platelet (PLT) count was 38–143 × 10^9^/L, and PLT transfusion was not indicated. Hemoglobin (HB) level declined to 95 g/L on the 4th day of life, so the infant received red blood cell (RBC) transfusion for the management of anemia. Blood test results suggested that the C‐reactive protein (CRP) level was <0.2 mg/L and procalcitonin (PCT) level was 0.25 ng/mL. Furthermore, the blood culture was negative. Serology test demonstrated negative results for toxoplasma, cytomegalovirus, rubella, herpes simplex virus I infections, herpes simplex virus II infections, and Human Parvovirus B19 DNA. Doppler echocardiography were examined, and PDA was left–right shunt with the diameter of 0.24 cm, Vmax was 2.1 m/s, and PG was 20 mmHg. Moreover, ultrasonography showed pleural effusion and seroperitoneum, without cardiac effusion (Figures 2 and 3). The clinical diagnosis of chylothorax was made based on the pleural fluid having elevated lymphocyte count (lymphocyte proportion >80%). Next‐generation sequencing (NGS) for pathogens of pleural effusion was negative. Whole‐exon gene sequencing of blood from the parents and amniotic fluid from the infant was performed.

## 3 CONCLUSION AND RESULTS

Whole‐exon gene sequencing of blood was trio‐based whole–exome sequencing. Whole‐exon gene sequencing of amniotic fluid showed one mutation of PTPN11 gene (NM_002834.5: c.922A > G (p.Asn308Asp)), heterozygous pathogenic variant, confirming the final diagnosis of NS. And de novo mutation was detected in this patient. The results of blood from the parents were negative. At last, her parents made a decision to withdraw medical care, and the newborn died after endotracheal extubation.

## 4 DISCUSSION

As a kind of genetic disease, NS is inherited in either the autosomal dominant or de novo manner.^4^ NS is usually related to lymphatic dysplasias, cardiac abnormalities, growth retardation and characteristic facies, as well as numerous additional developmental defects, such as mild‐to‐moderate developmental delay/learning disabilities, cryptorchidism in males, skeletal abnormalities, endocrine/metabolic imbalance, and predisposition to myeloproliferative diseases.^2^ NS patients are also associated with typical facial characteristics (namely, palpebral ptosis, hypertelorism, low‐set posteriorly rotated ears, downslanting palpebral fissures, low posterior hairline, or short/webbed neck), which are especially prominent in newborn period to middle childhood, whereas not that pronounced during the adulthood.^5^ As reported, 80% of NS patients exhibit congenital heart defects, as a result, it is the second most frequent syndromic cause leading to congenital cardiopathy following trisomy 21. Heart defects of high occurrence frequencies include pulmonary valve stenosis (PVS; 60%) and hypertrophic cardiomyopathy (HCM; 20%), nonetheless, numerous additional anomalies, like valve or septal defects, persistently patent arterial duct, and aortic coarctation, can also be observed.^6^ Fatal heart failure within 1 year old remains a concern among 1/4 of HCM patients.^3^

NS belongs to the RASopathy family, and dysregulation of Ras‐mitogen‐activated protein kinase (Ras/MAPK) pathway can be detected in this condition.^7^ Pathogenic variants of different components of this pathway are found to be associated with NS, with the frequently seen ones including PTPN11 (protein SHP‐2) (50%), SOS1 (10%–18%), RAF1, RIT1, KRAS, NRAS, BRAF, LZTR1, and SOS2.^2^, ^7^, ^8^ Ten or more different genes are identified to be associated with NS for the time being. NS may mainly result from the activation of mutations in genes that encode components of or regulating factors for RAS/MAPK pathway.^1^, ^8^ Typically, this pathway is ubiquitously activated upon various extracellular stimuli (such as hormones, growth factors, intercellular interaction) for modulating different cell activities (like proliferation, differentiation, migration, survival, and metabolism). Potent epidemiological evidence and mutation detection data are unavailable at present, but about 80% of NS patients may carry a mutation in such genes.^9^ Nonetheless, the pathogenic mutations have not been identified among 10%–20% of patients, besides, de novo mutations can be detected in most NS patients.^4^ Genetic heterogeneity may partially account for the difference in phenotype.^4^

There are a number of major clinical signs at each age that raise suspicion of NS. In the fetus stage, prenatal features can be frequently seen among NS fetuses, like persistent nuchal fold, elevated nuchal translucency, dilated jugular lymphatic sacs, hydrops fetalis, cystic hygroma, polyhydramnios, pleural or pericardial effusion, and cardiac abnormalities.^10^, ^11^, ^12^, ^13^ In the current case, the prenatal features included hydrops fetalis, pleural effusion, and polyhydramnios. The neonatal period stage is a pivotal period for diagnosing NS, however, NS may not be easily recognized at all time. Therefore, neonatologists must remember the characteristic facial features and implicit prenatal history of NS. The facial features of NS become less noticeable with age.^4^ Additional features are heart defects (in particular hypertrophic cardiomyopathy and pulmonary valve stenosis), feeding difficulties causing growth failure, and cryptorchidism in boys.^2^ In the childhood stage, monitoring of growth and developmental milestones provides opportunities for the identification of NS. The distinctive facial features of NS remain, and facial shape may become more triangular with age as the face lengthens. In addition, children with NS may present with skeletal abnormalities (pectus carinatum/pectus excavatum and scoliosis),^14^ bruising or bleeding,^15^ developmental delay/learning issues,^16^ and short stature,^17^ whereas feeding difficulties often resolve after the first years of life. In the case of short stature, children often have normal birth measurements but usually show a decline in their growth curve during the first years of life.^17^ In the adolescence stage, in addition to the characteristic skeletal, cardiac, bruising/bleeding, neurological and developmental features of NS, puberty may be delayed, particularly in boys, and the pubertal growth spurt may be absent.^17^ A study reported that more than 80% NS patients have some kind of heart malformation.^18^ The most common congenital heart disease is pulmonary stenosis accounting for 50%–60%, and followed by hypertrophic cardiomyopathy as the second common heart disease occurring in 20% of cases. The incidence of secundum atrial septal defect was 6%–10%. It is found that about 25% of patients die of heart failure due to secondary hypertrophic cardiomyopathy in the first year.^3^ Lymphatic vessel dysplasia or aplasia are present in less than 20% of infants with NS. The clinical symptoms include hydrops, congenital chylothorax, and peripheral lymphedema.^4^ Postnatally, the prevalence of lymphedema differs from 16% in patients with pathogenic PTPN11 variants to 44% in patients with pathogenic SOS1 variants, and the prevalence of acquired chylothorax is 4% in patients with pathogenic RIT1 variants.^19^ The types of lymphatic involvement that have been described in patients with NS include hydrops, chylothorax, pulmonary lymphangiectasis, anomalous lymphatic vessels in the thoracic cage, and aplasia or absence of the thoracic duct.^20^ A single‐center retrospective study had analyzed the central lymphoscintigraphy of 10 individuals with NS.^21^ They concluded that pulmonary lymphatic perfusion, retrograde intercostal lymphatic fow, and dysgenesis of the central lymphatic conduction system were characteristic performance of the children with NS. Gargano et al.^22^ also described a more severe phenotype including, hydrops fetalis, pleural effusions, and generalized edema resulting in death. Spontaneous early‐onset chylothorax prompting a diagnosis of Noonan syndrome has been reported in a few cases with severe pulmonary lymphangiectasis or significant respiratory distress at birth.^23^ The prevalence of hematologic abnormalities was reported to be 50%–89%.^24^ Coagulation defect occurs in a third of the patients with NS, including prolonged activated partial thromboplastin time (40%) and abnormalities of intrinsic pathway, other hematological abnormalities include thrombocytopenia, platelet function defects, mononucleosis, and myeloproliferative diseases.^25^ Some articles reported neonatal cases of NS who presented with refractory thrombocytopenia as the initial manifestation.^26^, ^27^

Despite the existence of diagnostic criteria, diagnosis is difficult in less severely affected infants.^2^ As recognition of the typical facial features is important in proposed diagnostic criteria, making the diagnosis especially difficult for someone who has little experience, some physicians may see patients with NS only rarely, and specialists treating individual manifestations of the syndrome in isolation may not be aware of their association with NS. The challenge is to remember this relatively rare diagnosis, which may present with a diverse range of symptoms that vary with severity. In some cases, the correct clinical diagnosis may be hampered by the presence of a more complex phenotype, for example the infant with hydrops and cardiomyopathy. There is currently no specific treatment for NS. Each patient requires an individualized management and has a different prognosis depending on symptoms and severity. As for any condition that involves multiple organ systems, a multidisciplinary team approach provides the best care for patients with NS.

**FIGURE 4:**
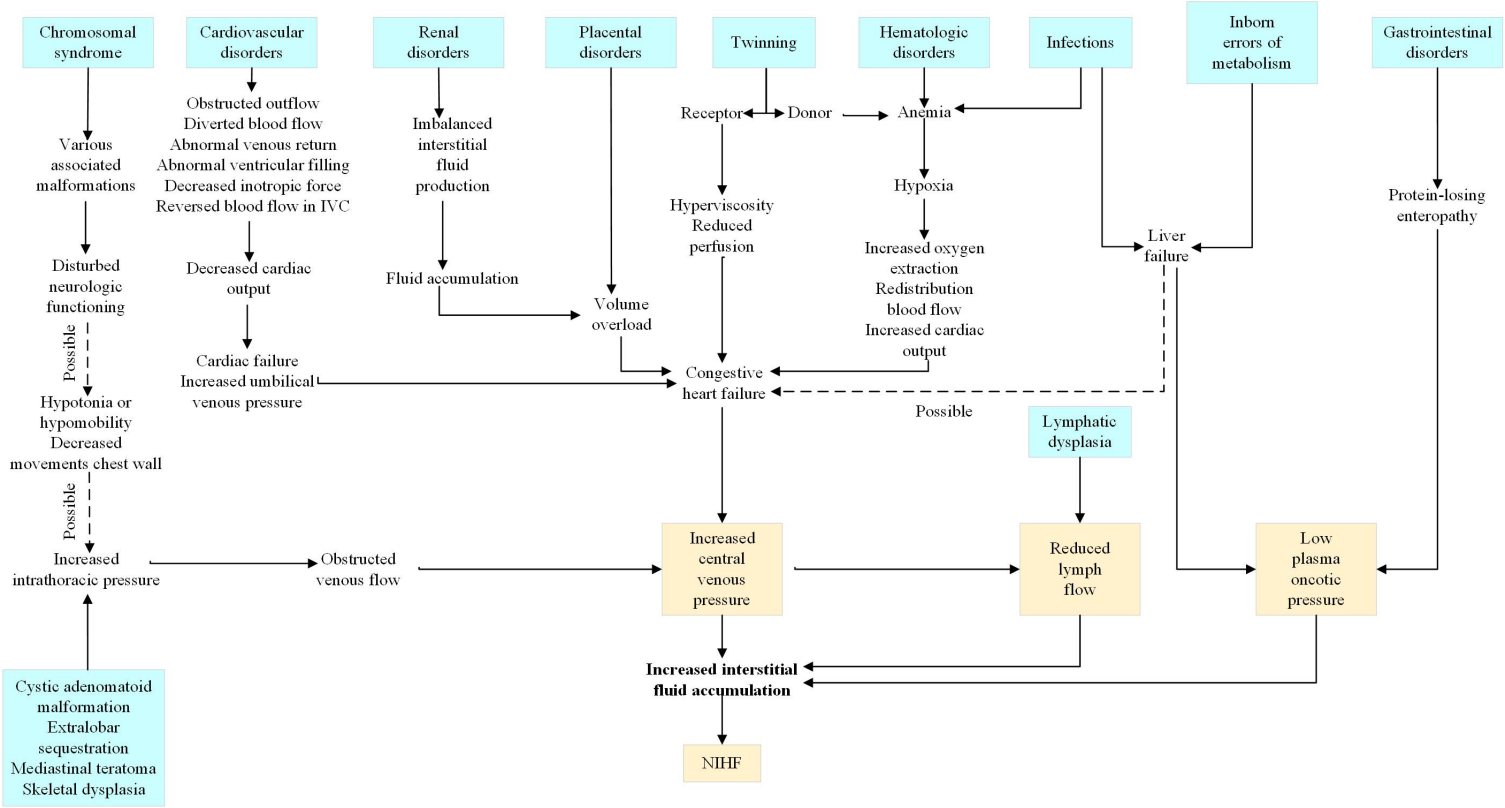
Various etiologies for nonimmune hydrops.^31^ IVC, inferior vena cava; NIHF, nonimmune hydrops fetalis.

Postnatal edema of the newborn is the continuation of hydrops fetalis. Nonimmune hydrops fetalis (NIHF) accounts for almost 90% of cases of hydrops.^28^ NIHF refers specifically to cases not caused by red cell alloimmunization. NIHF is the result of an increase in interstitial fluid production. Thus, hydrops fetalis should be seen as a symptom rather than as a disorder. The various causes of NIHF were classified into groups of disorders: cardiovascular disorders (21.7%), chromosome imbalances (13.4%), hematologic abnormalities (10.4%), infections (6.7%), intra‐thoracic masses (6.0%), lymph vessel dysplasias (5.7%), twin‐to‐twin transfusion syndrome and placental causes (5.6%), syndromes (4.4%), urinary tract malformations (2.3%), inborn errors of metabolism (1.1%), extra‐thoracic tumors (0.7%), gastrointestinal disorders (0.5%), miscellaneous causes (3.7%), and idiopathic (17.8%).^29^ A number of syndromes have been associated with NIHF, such as Noonan syndrome, multiple pterygium syndrome, Neu‐Laxova syndrome, hereditary lymphedema type 1, trisomy 13, trisomy 18, trisomy 21, and Turner syndrome.^30^ General pathophysiologic pathways to both prenatal and postnatal causes of NIHF is shown in Figure 4.^31^

After newborn resuscitation, the clinical management should address both the cause and the complications of hydrops. Morbidity and mortality may result from the hydropic state and the underlying causes. A fetus with hydrops that is delivered prematurely is subject to the additional complications of prematurity. If there is massive ascites or pleural effusions, initial resuscitation may require thoracentesis or peritoneal tap. Because of pulmonary edema, newborns with hydrops are susceptible to pulmonary hemorrhage and may require high levels of positive end‐expiratory pressure. Almost all of hydropic newborns will require mechanical ventilation because of pleural and peritoneal effusions, surfactant deficiency, pulmonary edema, poor chest wall compliance caused by edema, and persistent pulmonary hypertension of the newborn.^32^ The presence of persistent pleural effusions may necessitate the placement of chest tubes. Ascites may also compress the diaphragm and impair lung expansion. Preterm infants who need a prolonged course of ventilation may develop bronchopulmonary dysplasia. Management of lymphatic disorders typically includes furosemide to decrease pulmonary lymphatic flow. A low long‐chain fatty acid diet should also be used to reduce lymphatic fluid burden. Octreotide, a somatostatin analog that causes splanchnic vasoconstriction, has been used to treat patients with lymphatic flow disorders. Despite improvements in diagnosis and management, mortality from nonimmune hydrops remains high. For example, one study of 92 infants born with nonimmune hydrops between 2000 and 2012 reported a 45% fetal death rate and a 36% survival rate at 1 year.^33^ The best predictors of survival are the cause of the hydrops, the gestational age, and the condition of the neonate at birth. The lowest survival rates are for hydrops associated with a chromosomal diagnosis. A review of 598 patients with non‐immune hydrops found other risk factors for increased mortality, including younger gestational age, lower 5‐min Apgar score, and the need for increased respiratory support.^34^

End‐of‐life decisions are usually required when a neonate is at high risk of disability or death, and such decisions involve many legal and ethical challenges. Most neonatologists stated that limiting intensive care to “let nature take its course” can be ethically justifiable in terminal and fatal disease conditions.^35^ According to the data of the 1996 and 2016 surveys, withdrawing intensive care in the NICU as well as involving parents in decision makings have become more accepted, which could be as a result of administration of medical guidelines over the past two decades regarding joint decision‐makings for neonates at high risk of severe disability or death.^36^ Trend of physicians on how end the life of neonates, at risk of death, varies in different countries, and cultural factors, parents' involvement in decisions and gestational age are factors considered in end‐of‐life decision‐making.

With no family history, and an atypical phenotype, the clinical diagnosing of NS can be very difficult. The present case emphasized that NS should be considered in all fetuses with polyhydramnios, pleural effusions, and edema. Generalized edema in neonates may be the potential first symptom of NS.

## AUTHOR CONTRIBUTIONS

**Haifeng Zong:** Conceptualization; data curation; writing – original draft. **Yingsui Huang:** Resources. **Ying Xiong:** Data curation. **Wentao Gong:** Methodology. **Bingchun Lin:** Writing – review and editing. **Chuanzhong Yang:** Conceptualization; project administration; writing – review and editing.

## FUNDING INFORMATION

The present study was funded by the Shenzhen Fund for Guangdong Provincial high‐level Clinical Key Specialties (No. SZGSP009), the Sanming Project of Medicine in Shenzhen (SZSM201612045), and Shenzhen Science and Technology Innovation Committee (JCYJ20230807120201004 to HZ).

## CONFLICT OF INTEREST STATEMENT

The authors declare that there is no conflict of interest regarding the publication of this paper.

## ETHICS STATEMENT

This work gained approval from the Ethical Committee of Shenzhen Maternity and Child Healthcare Hospital.

## CONSENT

Written informed consent was obtained from the patient's parents to publish this report in accordance with the journal's patient consent policy.

## Data Availability

The data that support the findings of this study are available on request from the corresponding author. The data are not publicly available due to privacy or ethical restrictions.
